# Higher glucose level and systemic oxidative stress decrease the mean velocity index of the retinal artery during flickering light stimulation in type 1 diabetes

**DOI:** 10.3325/cmj.2016.57.434

**Published:** 2016-10

**Authors:** Vladimir Debelić, Brigita Drnovšek Olup, Bogomir Žižek, Milan Skitek, Aleš Jerin

**Affiliations:** 1 University Eye Clinic, University Medical Center in Ljubljana, Ljubljana, Slovenia; 2 Faculty of Health Sciences, University of Ljubljana, Ljubljana, Slovenia; 3 Institute for Clinical Chemistry and Biochemistry, University Medical Centre in Ljubljana, Ljubljana, Slovenia

## Abstract

**Aim:**

To determine whether higher glucose level and systemic oxidative stress decrease mean velocity (MV) index of the central retinal artery (CRA) during flickering light stimulation in type 1 diabetes (T1D).

**Methods:**

The study was performed in the period from 2008 to 2015 at the University Eye Clinic in Ljubljana. 41 patients with T1D and 37 participants without diabetes were included. MV in the CRA was measured using Doppler ultrasound diagnostics in basal conditions and during 8 Hz flickering light irritation. The plasma levels of glucose, fructosamine, 8-hydroxy-2'-deoxyguanosine (8-OHdG), triglycerides, cholesterol, and low-density lipoprotein (LDL) were measured.

**Results:**

Patients with T1D had significantly higher levels of blood glucose (*P* < 0.001), fructosamine (*P* < 0.001), and 8-OHdG (*P* < 0.001), but there were no significant differences in triglycerides (*P* = 0.108), cholesterol (*P* = 0.531), and LDL (*P* = 0.645) between the groups. Patients with T1D also had a significantly lower MV index in the CRA (1.11 ± 0.15 vs 1.24 ± 0.23; *P* = 0.010). In the T1D group, a significant negative correlation was found between the level of glucose (r = −0.58; *P* < 0.001), fructosamine (r = −0.46; *P* = 0.003), 8-OHdG (r = −0.48; *P* = 0.002) and the MV index in the CRA. At the same time, in this group fructosamine and 8-OHdG levels had a separate effect on the MV index (adjusted R^2^ = 0.38, *P* < 0.001).

**Conclusion:**

Higher glucose levels, the medium-term glucose level, and systemic oxidative stress could importantly reduce retinal vasodilatation during flickering light irritation in patients with T1D.

Metabolic changes caused by hyperglycemia in type 1 diabetes (T1D) affect retinal cells’ function, resulting in functional disturbances in the vessels during the increased metabolic load of the retina (1). Before changes are detected in the function of ganglion cells, there are malfunctions in neurovascular coupling, with modified vascular autoregulation in the retina (2). There are various diagnostic methods available to measure the extent of vasodilatation disability (3-5). Retinal irritation with flickering light causes increased metabolic activity, particularly in the ganglion retinal cells, with subsequent vasodilation of the local vessels and an increased blood flow. In this regard, an important role is played by vasodilators such as nitric oxide, as well as derivatives of arachidonic acid. The impact of some metabolites, such as lactate and adenosine diphosphate, was also identified (6,7). The vasoactive molecules are released directly from the nerve cells and neuroglia as well as the endothelial cells in the vessel walls (6,7).

Studies so far have discovered negative correlations between the retinal arterial dilatation during flickering light stimulation and blood glucose level 2 hours after oral glucose ingestion in patients with T1D (8) and in a healthy group (5). Retinal venous dilation in similar conditions was negatively correlated with glycated hemoglobin in patients with diabetes in one study (9), but another study did not show the same findings (8). The correlations between retinal vessel dysfunction and other systemic biochemical factors have not been investigated so far. Therefore, the aim of our study was to determine whether higher glucose and medium-term (1-2 weeks) glucose plasma level indicated by fructosamine, higher systemic oxidative stress indicated by 8-hydroxy-2'-deoxyguanosine (8-OHdG), and higher levels of triglyceride, cholesterol, and lipoproteins decrease mean velocity (MV) index of central retinal artery (CRA) in patients with T1D.

## Patients and methods

The study was carried out from July 2008 to January 2015 at the University Eye Hospital of the University Medical Centre in Ljubljana. All eligible patients with previously diagnosed T1D were participants in the diabetic retinal screening program at the outpatient department of the hospital. The patient group was randomly selected from a larger group of patients using random number generator. Healthy hospital employees were selected for the control group by the same technique from the list of employees taking into account approximately the same age and sex ratio compared to the patient group. The participants were invited to take part in the study and informed of the purpose and side effects of the research, and signed the informed consent form. Ethical approval was received from the Medical Ethics Committee of the Republic of Slovenia in the document number 38/02/07. To begin, a test sample size of 20 participants was assembled and the final size of both groups was determined by power and sample-size analysis for a two-sample means test. We needed a total number of 80 participants divided into two equal groups to detect the significant mean difference of the MV indices. The study involved 78 participants, who were divided into two approximately equal groups. Patient group comprised 41 patients with T1D and the control group comprised 37 participants without diabetes. By selecting patients with T1D, we avoided the effect of insulin resistance in type 2 diabetes (T2D), which could further affect the function of the vascular system. Also, patients with T2D more frequently suffer from obesity, arterial hypertension, hypertriglyceridemia, or lower high-density lipoprotein (HDL) levels (10), which might further affect the study results. In order to exclude the effect of age, the participants in both groups were of the same age (32.6 ± 6.7 years in the T1D group and 30.3 ± 5.1 years in the control group; *P* = 0.102). All the participants with untreated hypertension and a blood pressure of 140/90 mm Hg or more were not included in the study. Blood pressure was measured on the upper arm using a pressure monitor while the participants were sitting in an upright position. Individuals with a body mass index of more than 30 were also not included.

All the participants underwent an ophthalmologic examination of the anterior and posterior segments of the eye using a slit lamp, and the clinical documentation of patients with T1D was reviewed. The visual acuity with optical correction of all the participants was measured using a Snellen chart, and individuals with a VA of less than 20/25 were not included. Patients with T1D and any sign of diabetic retinopathy were also not included, as well as individuals with opaque optical media in the cornea, anterior segment, intraocular lens, and the vitreous body, and other retinal diseases (hypertensive retinopathy, occlusion of retinal vessels, etc). IOP was measured using Goldmann applanation tonometry. All patients with glaucoma and participants with an IOP of over 21 mm Hg were not included.

### Measuring the MV in the CRA using a Doppler ultrasound device

The MV was measured using the ATL HDI 1000 Ultrasound System (ATL Ultrasound Inc., Bothell, WA, USA), a Doppler ultrasound device, with a linear transducer 11-5 L. All the measurements were made from 09.00 am to 11:59 am by one person in a darkened room. Participants were allowed to have a five-minute rest period before the measurement and were lying in a horizontal position. The ultrasonic transducer was used to measure the MV in the CRA, just behind the eye in the optic nerve, through the upper eyelid with a fixed eye view at a 45-degree angle. In the first phase, three measurements were made in basal conditions, without light irritation, at five-minute intervals. In the second phase, the eyeball was exposed to 45 seconds of 8 Hz halogen lamp flickering light stimulation with a MV measurement made at the end of the irritation. Two similar measurements were repeated during five-minute intervals. The Pulsed-Wave Doppler Mode was used for a hemodynamic assessment of the MV in the CRA. The MV index is calculated from the ratio of two measurements:


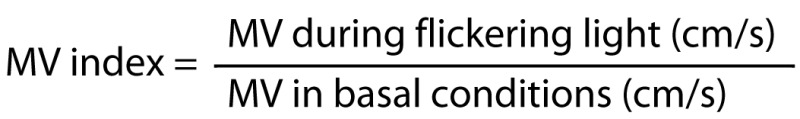
Equation 1

The measurements of the MV in the CRA in basal conditions were carried out by one person using a Doppler ultrasound device on 12 healthy participants in order to assess their repeatability. The measurements were carried out three times in three sessions, with one week between them.

### Biochemical analysis

Venous blood was taken from the cubital vein of the fasted participants, without using any additives, between 08.00 am and 09.00 am All the biochemical investigations were carried out at the Institute of Clinical Chemistry and Biochemistry of the University Medical Centre in Ljubljana. After blood centrifugation, the serum was stored at -20°C for further analyses. All the investigations were conducted simultaneously. Using enzymatic calorimetric methods, we determined the level of glucose, triglycerides, and cholesterol. Fructosamine was measured using the colorimetric method. HDL was measured using the direct method. The procedure was performed on an ADVIA automated analyzer (Siemens Diagnostics, Erlangen, Germany). Using the Friedewald formula, we determined the level of low-density lipoprotein (LDL) particles. The level of 8-OHdG in the serum was measured using the enzyme-linked immunosorbent assay method (JICA, Shizuoka, Japan).

### Statistical analysis

Normally distributed variables are presented as means and standard deviations and not-normally distributed variables are presented as medians and ranges. To assess the normality of distribution, we used the Shapiro-Wilk test. In both groups of participants, levels of cholesterol, HDL, LDL, and the MV values during the basal conditions were normally distributed. In patients with T1D, unlike the control group, fructosamine level and the MV index were normally distributed. In both groups, the levels of glucose, 8-OHdG and triglycerides, and the MV value during flickering light, were not normally distributed. The parametric variables were analyzed using an independent two-sample *t* test and the Pearson product moment correlation coefficient. For the nonparametric variables, we used the Mann-Whitney U-Test and the Spearman rank correlation coefficient test. Multiple linear regression was used to determine the influence of independent prognostic variables. We estimated the reproducibility of the measurements using the coefficient of variation. *P* values <0.05 were considered statistically significant. All statistical calculations were performed using IBM SPSS Statistics v. 20 software (IBM, Armonk, NY, USA).

## Results

There were no differences in sex (18 men and 23 women in the T1D group vs 16 men and 21 women in the control group; *P* = 0.963) and age between the groups (32.6 ± 6.7 years and 30.3 ± 5.1 years, respectively; *P* = 0.102). The patients had T1D for 18.1 ± 7.9 years. 15 patients had an insulin pump, while the rest were treated with insulin injections. 5 patients with T1D and only one control participant were treated for hypertension before the study, but the difference was not significant (*P* = 0.124). Systolic (SBP)/diastolic blood pressure (DBP) was lower than 140/90 mm Hg in all participants. There were no significant differences between the groups in either SBP (T1D group 117.7 ± 12.7 mm Hg and controls 119.7 ± 12.4 mm Hg, *P* = 0.480) or DBP (74.2 ± 8.4 mm Hg and 72.2 ± 8.9 mm Hg, respectively, *P* = 0.308). 2 control participants and 8 patients with T1D were smokers, but the difference in smoking status between the groups was not significant (*P* = 0.063).

The IOP was approximately the same in both groups (15.8 mm Hg ±3.4 in T1D group vs 15.0 ± 5.4 mm Hg in controls; *P* = 0.424). Patients with T1D had significantly higher average levels of glucose (*P* < 0.001), fructosamine (*P* < 0.001), and 8-OHdG (*P* < 0.001). However, there were no significant differences in the levels of triglycerides, cholesterol, LDL, and HDL (Table 1). 58.5% of patients with T1D and 5.4% of controls had fasting glucose level higher than 6.1 mmol/L.

**Table 1:** Biochemical analyses in patients with type 1 diabetes and control group

Variable	Type 1 diabetes (n = 41)	Control group (n = 37)	*P*
Glucose (mmol/L)	7.3 (4.4-8.6)*	4.0 (3.3-4.5)	<0.001†
Fructosamine (mg/L)	392 ± 103	217 (189-252)	<0.001†
8-hydroxy-2'-deoxyguanosine (µg/L)	28.08 (22.13-35.82)	12.43 (6.35- 22.25)	<0.001†
Cholesterol (mmol/L)	4.7 ± 0.7	4.8 ± 0.9	0.531
High density lipoproteins (mmol/L)	1.4 ± 0.3	1.4 ± 0.3	0.793
Low density lipoproteins (mmol/L)	2.7 ± 0.7	2.6 ± 0.7	0.645
Triglyceride (mmol/L)	0.9 (0.7-1.1)	1.0 (0.8-1.3)	0.108†

*Mean ± standard deviation for normally and median (interquartile range) for not-normally distributed variables.

†Mann-Whitney U-test; unmarked independent two-sample *t* test.

In patients with T1D, there were significant positive correlations between the level of glucose and fructosamine (r = 0.80; *P* < 0.001) and the level of glucose and 8-OHdG (r = 0.45; *P* = 0.003). Similarly, in the control group there was a positive correlation between the level of glucose and fructosamine (r = 0.51; *P* = 0.001) but there was no correlation between the level of glucose and 8-OHdG (r = 0.23; *P* = 0.143). Multiple linear regression showed that fructosamine and 8-OHdG levels had a separate effect on the MV index in patients with T1D. The regression model explained the greatest proportion of the variability of the MV index (R^2^ of the model = 0.38; *P* < 0.001) (Table 2).

**Table 2:** Model of multiple linear regression for analyzing the influence of different independent variables on the mean velocity index in patients with type 1 diabetes*

Variable	b	*P*	R^2^ of the sequence	R^2^ of the model
8-hydroxy-2'-deoxyguanosine	−0.0047	<0.001	0.28	0.38
Fructosamine	−0.00048	0.013	0.38	*P* < 0.001

*b − regression coefficient; R˛ − coefficient of determination.

Patients with T1D had significantly lower average MV in the CRA in basal conditions (*P* = 0.019) and during flickering light stimulation (*P* < 0.001). The MV index was also significantly lower in that group (*P* = 0.010) (Table 3).

**Table 3:** The mean velocity (MV) in the central retinal artery and the MV index in patients with type 1 diabetes and the control group

Variable	Type 1 diabetes (n = 41)	Control group (n = 37)	*P*
MV in basal conditions (cm/s)	6.44 ± 1.70	7.44 ± 1.99	0.019
MV during flickering light (cm/s)	6.8 (5.83-7.75)	9.10 (7.41-10.56)	<0.001†
MV index	1.11 ± 0.15	1.17 (1.08- 1.36)	0.010†

*Mean ± standard deviation for normally and median (interquartile range) for not-normally distributed variables.

†Mann-Whitney U-Test; unmarked independent two-sample *t* test.

The repeatability of the three measurements was tested on 12 participants. The coefficient of the variation, or repeatability, of measurements of the MV was 5.6%. The correlation coefficient between the measurements of this parameter by the same investigator was 0.89 (*P* < 0.001).

**Figure 1:**
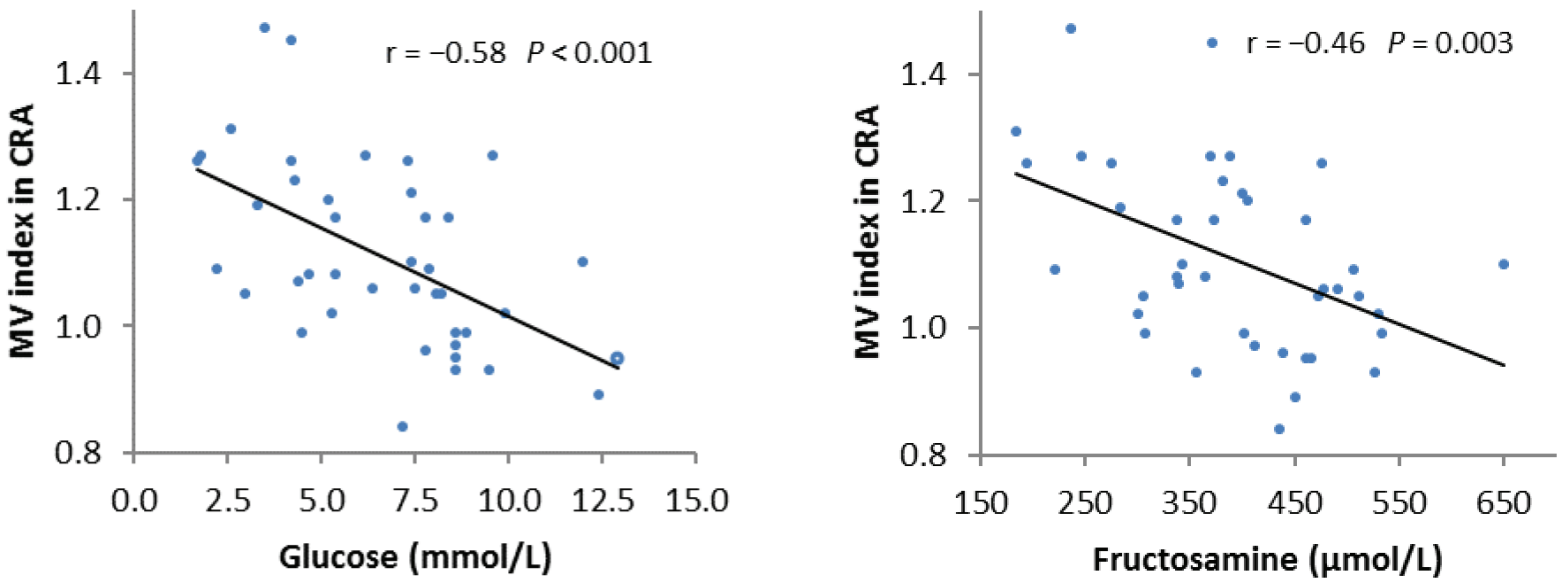
Correlation between the level of glucose/fructosamine and the mean velocity (MV) index in the central retinal artery (CRA) in patients with type 1 diabetes (T1D); r – coefficient of correlation.

**Figure 2:**
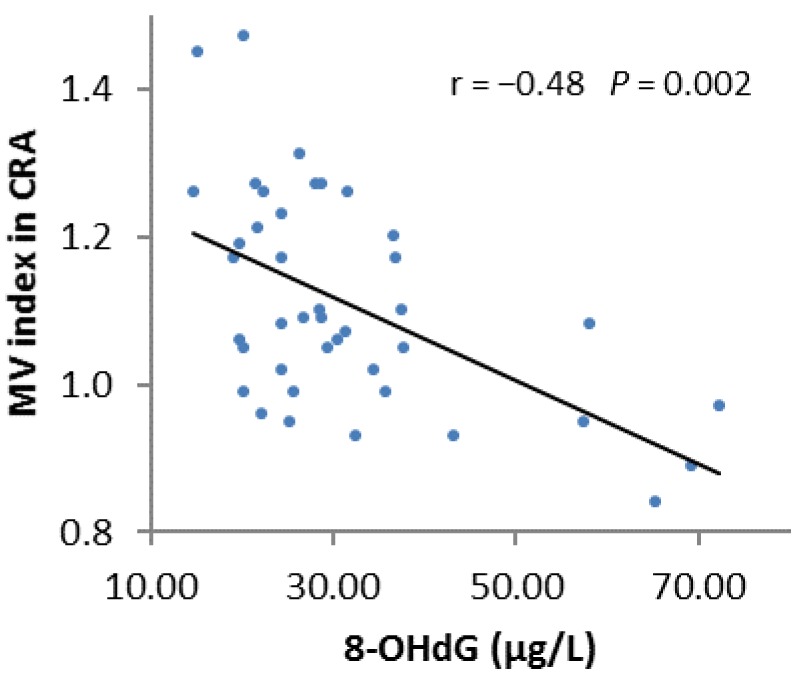
Correlation between 8-hydroxy-2'-deoxyguanosine level and the mean velocity (MV) index in the central retinal artery (CRA) in patients with type 1 diabetes (T1D); r – coefficient of correlation.

In patients with T1D, a significant negative correlation was found between the MV index and the level of glucose (r = −0.58; *P* < 0.001), fructosamine (r = −0.46; *P* = 0.003), and 8-OHdG (r = −0.48; *P* = 0.002). In the control group, there was a comparable significant positive correlation between the MV index and the fructosamine level. In patients with T1D, there was no significant correlation between participants’ age, duration of diabetes, SBP, DBP, IOP, cholesterol levels, HDL, LDL, and triglycerides with the MV index (Table 4 and Figures 1 and 2).

**Table 4:** Correlation between mean velocity index and clinical or biochemical variables in patients with type 1 diabetes and the control group*

	Type 1 diabetes (n = 41)		Control group (n = 37)	
Variable	r	*P*	r	*P*
Age	0.045	0.781	0.0081†	0.962
Diabetes duration	0.17	0.294		
SBP	−0.092	0.572	0.0017†	0.995
DBP	0.15	0.362	0.11†	0.514
IOP	0.27	0.083	−0.14†	0.408
Glucose	−0.58†	<0.001	0.23†	0.166
Fructosamine	−0.46	0.003	0.33†	0.045
8-OHdG	−0.48†	0.002	0.18†	0.276
Cholesterol	−0.12	0.471	0.40†	0.014
HDL	−0.10	0.521	0.29†	0.087
LDL	0.00010	0.984	0.14†	0.403
Triglyceride	−0.16†	0.315	−0.18†	0.293

*SBP – systolic blood pressure; DBP – diastolic blood pressure; IOP – intraocular pressure; 8-OHdG – 8-hydroxy-2'-deoxyguanosine; HDL – high density lipoproteins; LDL – low density lipoproteins; r – coefficient of correlation.

†Spearman rank correlation coefficient; unmarked Pearson product moment correlation coefficient.

## Discussion

Using a Doppler ultrasound examination on Pulsed-Wave Doppler Mode, we observed lower ability of retinal vasoregulation during increased retinal metabolic load in patients with T1D. Similar results using the same technique have not been published in diabetic patients recently but lower retinal vasodilation in similar conditions was found using another technique (Dynamic Vessel Analyzer) (11,12). Our results indicate that diabetes has a significant impact on the reduction of vasodilatation during increased retinal metabolism due to flickering light stimulation. Neurodegenerative changes in the retina occur early in the course of diabetes and affect neurovascular coupling. In particular, there are interferences in the release of the vasodilators when metabolic requirements are high (6,13,14). Direct vascular failure in diabetes takes place only afterwards (2).

An ultrasound examination using the Pulsed-Wave Doppler Mode provides a high degree of precision, with appropriate reproducibility, for measuring blood velocity in the orbital vessels and corresponds to the intra-subject reproducibility of our measurements of the MV in the CRA (15,16). Taking these results into account, we can conclude that the method is reliable for measuring the blood flow in the CRA and that it can be used for further investigations.

In appropriately controlled and treated patients with diabetes, the fasting glucose level in the morning should be in the normal range. In insulin-treated patients with T1D we found higher plasma glucose level than in controls, and in more than half of the patients it was above the upper limit of the normal range. The majority of our patients had poor glycemic control. Patients with T1D with hyperglycemia 1 and 2 h after glucose ingestion and no clinical signs of diabetic retinopathy had a significantly lower retinal vasodilatation ability during flickering light stimulation when measured by the Retinal Vessel Analyzer but after normalization of hyperglycemia, the vasodilatation ability quickly returned to normal (8). Using the same measuring and stimulation technique, an increased blood glucose level in healthy people was found to have a negative effect on retinal vasodilatation (5). When Doppler ultrasound device was used in our study, a negative correlation was recorded between the glucose level and the MV index in the CRA in patients with T1D. There was evidence of the impact of blood glucose on retinal blood vessel dysfunction. Hyperglycemia instigates biochemical changes that play an important role in the development of retinal neuronal and vascular dysfunction (17).

Fructosamines result from the glycation of serum proteins and reflect the mean blood glucose levels for the previous two to three weeks, which can be used as clinical indicators of recent changes in glycemic control (18). The link between the glycated hemoglobin level and retinal vasodilatation ability during flickering light stimulation is described in the literature, but no reference is made to the links with fructosamine (8,19). We revealed for the first time a significant negative correlation between fructosamine level and the MV index in the CRA during retinal flickering light irritation in patients with T1D. This result confirms that the impact of the higher medium-term glucose level in patients with T1D also has as important an effect on retinal vasodilatation as the current blood glucose level.

8-OHdG levels in patients with diabetes are higher than in control participants, not only in the blood but also in the urine and vitreous body (20,21). As was expected, we found a similar difference in the plasma 8-OHdG levels. 8-OHdG is an indicator of oxidative stress and is formed by oxidation of the deoxyguanosine in the DNA in the nucleus and mitochondria. Increasing amounts of ROS are found in the serum of diabetic patients, especially in those with poor glycemic control. The relative increase in oxidative metabolites contributes to the damage of retinal vessels (22). Damage to mitochondrial DNA has a negative impact on the metabolism and leads to a dysfunction, particularly of the ganglion and retinal vascular cells (2). The influence of an oxidative stress marker or ROS component on retinal vasodilatation ability due to flickering light stimulation in patients with diabetes is not described in the literature. In the T1D group, we found an inverse relationship between the 8-OHdG level and the MV index in the CRA during flickering light stimulation owing to the increased metabolic load. Due to the positive interdependence between the level of glucose and 8-OHdG, the question arose as to whether increased oxidative stress might be a direct cause of worsened vasodilatation or simply a by-product of glucose metabolism. We found a statistically independent effect of 8-OHdG level on this index in patients with T1D. These findings indicate that higher systemic oxidative stress or individual components of ROS in the blood could directly affect the malfunction of the retinal vasculature during increased metabolic load due to irritation caused by flickering light. Oxidative stress is the consequence of hyperglycemia, which in turn causes the overproduction of ROS in body fluids.

Hypertension and T1D coexist more frequently than usual (23), but we did not observe a higher frequency of hypertension in patients with T1D. We also did not find an effect of normal blood pressure on the change of the MV index in the CRA during flickering light stimulation.

Diabetic patients with a normally controlled disease usually have normal or suppressed levels of triglycerides and LDL particles, while patients with poorly controlled glycemia commonly have elevated triglycerides and LDL levels (24). In this study, both groups had lipid levels within normal range, and there were no differences between the groups.

The importance of the study is increased by the high reproducibility of measurements, which is manifested as a low coefficient of variation and a high positive correlation coefficient between the individual measurements taken from a certain number of patients. This method enables the rapid detection of dysfunction in morphologically unchanged retinal vessels. There have not yet been any similar comparative studies among younger people with T1D.

There are several limitations of this study. We did not measure the CRA diameter in the optic nerve concomitantly with MV. Our result allowed only partial description of the retinal hemodynamic properties and it was not possible to calculate the blood flow in the retinal vessels. The levels of fructosamine and 8-OHdG level in our study were not comparable with the results of other studies because these measurements were used only for research purposes and there are no general reference values for comparison of the results. 5 patients with T1D and only 1 man in the control group were treated for arterial hypertension before the beginning of the study. Antihypertensive drugs and treated arterial hypertension could affect the dysfunction of the retinal blood vessels during the flickering light irritation and the accuracy of measurements of MV in CRA.

The significance of the functional vascular disorder of the retina during flickering light stimulation in the formation and prognosis of diabetic retinal pathology is not yet known. Despite the observed connection between systemic oxidative stress and functional vascular disorders of the retina, measurement of the 8-OHdG level during screening of the patients with T1D has no clinical significance. Further studies are required to identify possible connections between retinal functional vascular disorders and clinically undetectable morphological changes in the retina, the probability of occurrence and rate of progression of diabetic retinopathy, and the possibility of severe and proliferative diabetic retinopathy. If these hypotheses are accepted, the measurement of retinal functional vascular disorder, and even more systemic biochemical markers associated with it, will play an important role in long-term screening of the patients with T1D.
